# Three-Dimensional CT Findings of Os Calcaneus Secundarius Mimicking a Fracture

**DOI:** 10.1155/2014/537062

**Published:** 2014-12-21

**Authors:** Mehmet Deniz Bulut, Alpaslan Yavuz, Aydın Bora, Mehmet Ata Gökalp, Sercan Özkaçmaz, Abdussamet Batur

**Affiliations:** ^1^Department of Radiology, School of Medicine, Yuzuncu Yil University, Dursun Odabaş Medical Center, 65100 Van, Turkey; ^2^Department of Orthopedics, School of Medicine, Yuzuncu Yil University, 65100 Van, Turkey

## Abstract

Os calcaneus secundarius is one of several accessory ossicles of the foot that have been identified as normal variants of skeletal development. It may cause ankle pain and may mimic an avulsion fracture of the anterior calcaneal process. A twenty-year-old male was admitted to our institution with right ankle pain following an inversion injury. An axial CT image of the patient's right ankle revealed a shape with smooth and sharp margins, identified as a well-corticated bone fragment in the subtalar region. A diagnosis of an accessory ossicle, os calcaneus secundarius, was made based on radiographic findings. As a result of this case, it is recommended that potential locations of the accessory bones should be well understood in order to prevent misdiagnosis and inappropriate surgical procedures. Os calcaneus secundarius must be considered when an apparent bone fragment or a suspicious fracture line at the anterior region of os calcaneus is demonstrated.

## 1. Introduction

Accessory ossicles of the foot occurred as normal variants of skeletal development [1]. They are thought to occur either due to the separation of a single center or through a failure in the union of ossification sites [2]. An accessory ossicle may be located adjacent to the originating bone or may be identified as a separate bone [3]. Accessory bones are mostly asymptomatic and are detected incidentally by radiological examinations. Os calcaneus secundarius, which is such an accessory bone, may cause ankle pain and may also mimic an avulsion fracture of the anterior calcaneal process. To our knowledge, four cases of os calcaneus secundarius have been reported in the literature as three were identified by plain radiographs and one was identified with volume rendering images. We presented this case because of the limited radiologic description of the entity by the recent literature.

## 2. Case Report

**Figure 1:**
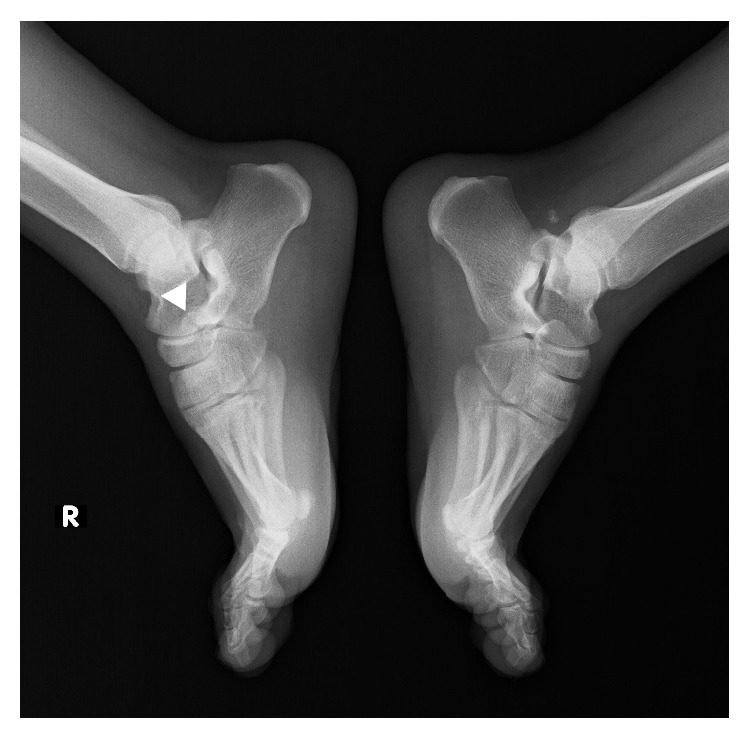
Lateral plain radiograph shows a suspicious fracture line of anterosuperior calcaneal process (white arrowhead).

**Figure 2:**
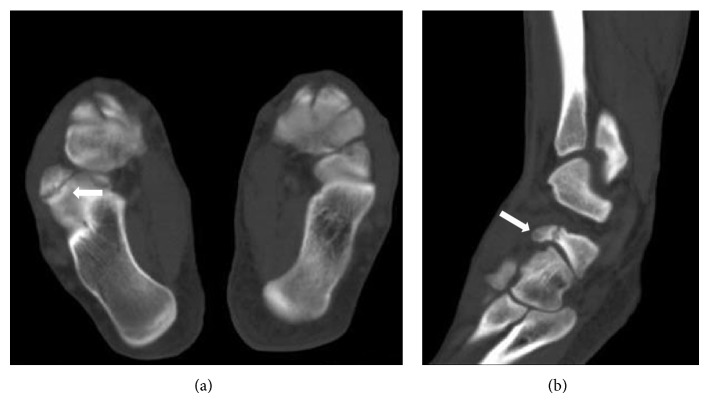
Axial (a) and sagittal (b) reformatted CT images reveal os calcaneus secundarius (white arrows).

**Figure 3:**
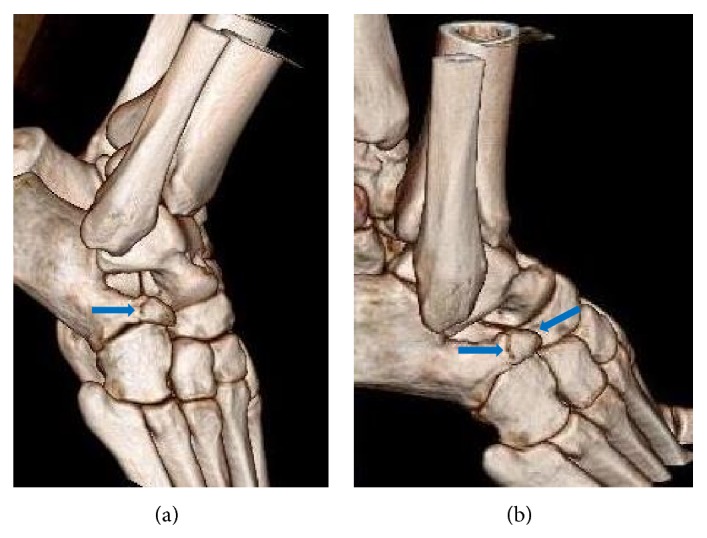
3D volume rendering CT images (a and b) demonstrate os calcaneus secundarius (blue arrows).

A twenty-year-old male was admitted to our institution complaining of right ankle pain after an inversion injury. On physical examination, swelling and tenderness over the calcaneocuboid joint were detected. Anteroposterior and lateral radiographs of the right ankle showed a suspicious fracture line on the anterior process of the calcaneus and what appeared to be a bone fragment between the calcaneus and the cuboideum. Comparative consideration of the contralateral ankle based on plain radiographs did not demonstrate any similar findings, only a fracture line on anterior aspect of the right talus could suspiciously be determined (Figure 1). An advanced radiologic examination was then performed, consisting of a computed tomography (CT) scan of the right ankle with axial (Figure 2(a)), multiplanar (Figure 2(b)), and three-dimensional (3D) volume rendering (Figure 3). The CT images revealed a smoothly and sharply marginated well-corticated bone fragment in the subtalar region. With these findings, a diagnosis of os calcaneus secundarius was made. The patient was treated with the administration of nonsteroidal anti-inflammatory drugs and the symptoms were evidently relieved within the following 10 days. Finally, he was free of pain during the mobilization after 30 days.

## 3. Discussion

Most accessory ossicles tend to remain asymptomatic and cause no complaints. They are frequently detected incidentally on radiological examinations that are performed because of a complaint of pain that is secondary to trauma or to degenerative changes due to overuse [3, 4]. Os calcaneus secundarius is an accessory ossicle of the anterior facet of the calcaneus and is encountered in up to 5% of the population [5]. Os calcaneus secundarius was first described by Stieda in 1869, and the earliest known specimen of a calcaneus secundarius was reported by Holland in 1928 [6, 7]. The main location of this ossicle, which stands on the dorsal projection of the calcaneus, is the gap between the anteromedial aspect of the os calcis, the proximal aspect of the cuboid and navicular, and the head of the talus [8].

These ossicles may mimic an avulsion fracture, especially if there is a coincident traumatic injury to the anterior process of the calcaneus [9]. Differentiation of os calcaneus secundarius and fracture has a clinical significance, as the management protocols of these two facts are very distinct. Moreover, the differential diagnosis of these entities on the sole basis of plain radiographs and physical examination can be challenging [10]. Lateral and oblique views are the most useful plain radiographs in calcaneus secundarius and anterior process fracture detection [11]. CT scans can simply facilitate the diagnosis of an accessory ossicle by clearly demonstrating a smoothly and sharply margined well-corticated bone [12]. In our case, although physical examination and plain radiographs suggested a fracture of the anterior process of the calcaneus, a final diagnosis of os calcaneus secundarius could become evident after a CT scan.

In conclusion, clinicians should fully understand the localizations of accessory bones in order to avoid misdiagnosis and improper invasive procedures. We suggest consideration of os calcaneus secundarius when a suspicious fracture line at the anterior part of the calcaneus and a bone fragment are detected simultaneously. We further recommend performing CT scans in similar cases to ensure an accurate diagnosis.
